# The Clinical Outcomes of Preoperative Self‐Expandable Metal Stent Placement as Bridge to Surgery for Obstructive Colorectal Cancer

**DOI:** 10.1002/cnr2.70255

**Published:** 2025-06-16

**Authors:** Ryosuke Aoki, Nao Hondo, Masato Kitazawa, Satoshi Nakamura, Makoto Koyama, Masahiro Kataoka, Hirokazu Tanaka, Takuya Iguchi, Yonghan Park, Yuji Soejima

**Affiliations:** ^1^ Division of Gastroenterological, Hepato‐Biliary‐Pancreatic, Transplantation and Pediatric Surgery, Department of Surgery Shinshu University School of Medicine Nagano Japan

**Keywords:** bridge to surgery, long‐term outcomes, obstructive colorectal cancer, self‐expandable metal stents, short‐term outcomes

## Abstract

**Background:**

Self‐expandable metal stent (SEMS) placement for obstructive colorectal cancer (OCRC) is widely performed as a bridge to surgery because of its lower mortality rate than emergency surgery. However, the long‐term outcomes remain unclear because of the risk of complications and cancer recurrence. This study investigated the short‐ and long‐term outcomes of SEMS placement for OCRC.

**Methods and Results:**

We retrospectively reviewed the clinicopathological data of patients with OCRC who underwent preoperative treatment and tumor resection at our institution between April 2004 and March 2022. Among 113 patients with OCRC, 30 underwent SEMS placement (Group S) and 36 underwent ileus tube insertion (Group T); Group S had more older patients and fewer pT4 cases; no other characteristics differed. Incidence of severe complications was 0% versus 16.7% (*p* < 0.01); postoperative hospital stay was 14.3 versus 26.6 days (*p* < 0.01); medical costs were comparable between the groups. The long‐term outcomes of Group S and patients with non‐OCRC who underwent surgery during the same period were compared after propensity score matching. The 5‐year survival rates were 69.5% and 77.1%; the 5‐year recurrence‐free survival rates were 44.5% and 55.5%, without significant difference.

**Conclusion:**

In conclusion, SEMS placement was more effective than ileus tube placement in treating OCRC.

## 1 Introduction

Colorectal obstruction by colorectal cancer is a medical emergency requiring urgent decompression procedures. At initial diagnosis, 7%–16% of patients with colorectal cancer already exhibit symptoms of intestinal obstruction [1]. Common treatments for decompression are emergency surgery, ileus tube insertion, and stent placement. Emergency surgery is often associated with high rates of mortality and complications and typically requires colostomy or ileostomy [2].; thus determining ways to avoid emergency surgery and relieve intestinal obstruction is important. In recent years, self‐expandable metal stent (SEMS) placement has gained attention as a bridge to surgery (BTS) to avoid emergency surgery. Short‐term outcomes suggest that SEMS placement is superior to emergency surgery, and the 2020 European Society of Gastrointestinal Endoscopy guidelines recommend SEMS for obstructive colorectal cancer (OCRC) to avoid emergency surgery [3]. However, SEMS placement has risks such as perforation, stent migration and re‐obstruction, which may affect overall survival [4]. In contrast, some reports suggest that SEMS placement does not affect long‐term prognosis [5, 6]. Although it seems prudent to avoid emergency surgery in the management of OCRC, the choice between SEMS placement and decompression tube insertion as a BTS treatment remains controversial in daily clinical practice. Additionally, transanal tubes are relatively inexpensive, ranging from USD 300 to 480, whereas SEMS are expensive, priced at USD 1700 in Japan. Thus, choosing an appropriate BTS strategy required consideration of both clinical outcomes and medical costs. In this study, we aimed to retrospectively examine the short‐ and long‐term outcomes of SEMS placement in patients with OCRC at our institution.

## 2 Materials and Methods

### 2.1 Data Sources and Selection Criteria

This retrospective, observational, single‐center study was performed at Shinshu University School of Medicine between April 2004 and March 2022. This study included 1046 patients with colorectal cancer who underwent primary tumor resection at our hospital. The study protocol for this research project was approved by the Institutional Ethics Committee of Shinshu University and conformed to the provisions of the Declaration of Helsinki (approval number: 5691). Two experiments were conducted in this study.

### 2.2 Study 1

**FIGURE 1:**
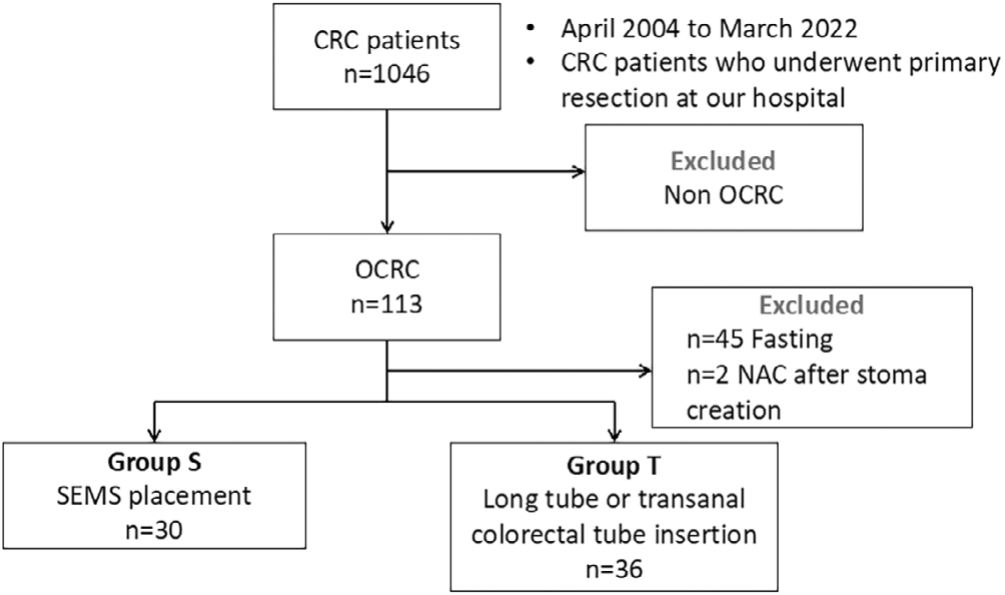
Flow chart of population selection of study 1. Abbreviations: CRC, colorectal cancer; NAC, neo‐adjuvant chemotherapy; OCRC, obstructive colorectal cancer; SEMS, self‐expandable metal stent.

Among the 1046 cases, we excluded 933 without signs of malignant colorectal obstruction, and the study included a cohort of 113 patients. Of these 113 patients, 45 who were treated with fasting alone and two who were treated with emergency stoma creation surgery followed by neoadjuvant chemotherapy were excluded. Of the remaining 66 patients, 30 underwent SEMS placement (Group S) and 36 underwent ileus tube insertion (Group T) (Figure 1). The short‐term perioperative outcomes were compared between groups S and T. The total medical cost, including SEMS placement, was calculated from medical fee points.

### 2.3 Study 2

**FIGURE 2:**
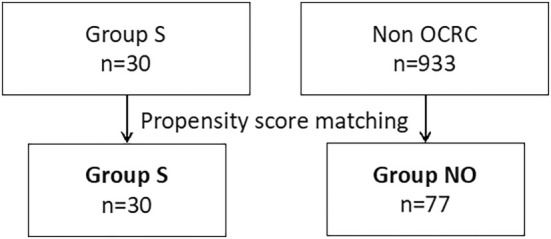
Flow chart of population selection of study 2. OCRC, obstructive colorectal cancer; Group S, patients who underwent self‐expandable metal stent placement; Group NO, patients selected using propensity score matching.

We compared the long‐term prognosis of Group S with that of 933 patients with non‐obstructive colorectal cancer among 1046 cases. One to three propensity score matching (PSM) analyses were performed to reduce selection bias. Propensity scores were estimated using a generalized linear model based on sex, age, performance status (PS), tumor location, and pathological stage. Patients selected using PSM were assigned to Group NO (non‐obstructive) (Figure 2). The long‐term outcomes were compared between Group S and Group NO.

### 2.4 SEMS Placement or Ileus Tube Insertion

Patients presenting symptoms such as abdominal pain, abdominal distension, nausea, vomiting, and cessation of bowel movements or gas underwent computed tomography (CT) upon admission to our hospital. When colorectal cancer‐induced obstruction was suspected, lower gastrointestinal endoscopy was performed by gastroenterology internists at our hospital. If a tumor‐related obstruction is identified, a definitive diagnosis is made through histological biopsy, and measures for relieving the obstruction are concurrently considered. Since SEMS placement became eligible for insurance coverage, SEMS (Niti‐S) is our hospital's first choice of treatment, whenever possible, to comprehensively assess the convenience of preoperative management and patient burden. In cases of ileus tube insertion, intermittent irrigation is performed while monitoring the progress through abdominal plain radiography or CT. Once the relief of intestinal obstruction is confirmed and the condition stabilizes, surgical treatment is initiated. Once patients who underwent SEMS placement, demonstrated the ability to ingest food and had stable bowel movements, they were discharged from the hospital, and elective surgery was scheduled.

### 2.5 Statistical Analyses

Statistical analyses were conducted using the EZR [7]. Numerical variables were evaluated using the Mann–Whitney *U* test, and categorical variables were evaluated using Fisher's exact probability test. Overall survival and recurrence‐free survival are presented as survival curves plotted using the Kaplan–Meier method and compared using log‐rank tests and the Cox proportional hazards model. Two‐sided *p* < 0.05 were considered statistically significant.

## 3 Results

### 3.1 Study 1

The clinicopathological characteristics of Groups S and T are presented in Table 1. The mean age was 72.2 years in Group S, and it was significantly older than that in Group T (*p* = 0.03). No significant differences in sex, PS, or tumor location were observed between the two groups. Mean CROSS scores before decompression were also comparable. In the examination of pathological factors, Group S had 21 pT3 and 9 pT4 cases and Group T had 13 pT3 and 23 pT4 cases. Group T had significantly more pT4 cases (*p* = 0.01). There were no significant differences in other *N* factors, the presence of distant metastasis, or pathological stage.

**TABLE 1:** Patients' characteristics.

	Group S, *n* = 30	Group T, *n* = 36	*p*
Age, mean ± SD	72.2 ± 2.7	64.9 ± 2.2	0.03^a^
Male, *n* (%)	20 (66.7)	25 (69.4)	1.00
PS, 0/1/2, *n*	25/5/0	27/6/3	0.35
Site, A/T/D/S/R	1/5/2/14/8	7/3/1/12/13	0.30
CROSS score, mean ± SD	1.4 ± 0.77	1.42 ± 0.94	0.94
pT, 3/4, *n*	21/9	13/23	0.01^a^
pN, 0/1/2/3, *n*	12/9/8/1	12/12/9/3	0.87
pM, 0/1, *n*	17/13	16/20	0.46
pStage, II/III/IV, *n*	10/7/13	7/9/20	0.47

*Note:* Data are given as mean ± standard deviation or *n* (%).

Abbreviations: A, ascending colon; D, descending colon; PS, performance status; R, rectum; S, sigmoid colon; T, transverse colon.

^a^ Significant difference.

Perioperative outcomes are summarized in Table 2. In each group, two cases of perforation were observed during the waiting period from preoperative treatment to surgery, but there was no significant difference. The time from decompression to surgery was longer in Group S, although the difference was not statistically significant (34.0 vs. 14.8 days). The rate of laparoscopic surgery was 40% in Group S and 2.7% in Group T, with significantly more laparoscopic surgeries performed in Group S (*p* < 0.01). The blood loss was 286.6 mL in Group S and 735.7 mL in Group T, with significantly less bleeding in Group S (*p* = 0.03). Although there was no significant difference in complications of all grades according to the Clavien–Dindo classification, severe complications of grade ≥ 3 were significantly fewer in Group S (0% vs. 16.7%, *p* < 0.01). In Group T, grade ≥ 3 complications included one case each of common bile duct stone, pleural effusion, sepsis from central venous catheter infection, anastomotic leakage, and two cases of ileus. Owing to fewer complications, the postoperative hospital stay was significantly shorter in Group S than in Group T (14.3 vs. 26.6 days, *p* < 0.01). There was no significant difference in overall medical costs between the two groups.

**TABLE 2:** Perioperative outcomes.

	Group S, *n* = 30	Group T, *n* = 36	*p*
Perforation, *n* (%)	2 (6.7)	2 (5.6)	1.00
Interval between decompression and surgery, days	34 ± 55.7	14.8 ± 11.8	0.07
Laparoscopic surgery, *n* (%)	12 (40)	1 (2.7)	< 0.01^a^
Stoma creation, *n* (%)	7 (23.3)	14 (38.9)	0.20
Operation time, min	326.6 ± 18.2	359.5 ± 33.5	0.74
Blood loss, mL	286.6 ± 59.8	735.7 ± 189.3	0.03^a^
All grade complications, *n* (%)	17.0 (56.7)	13.0 (40.0)	0.14
Gr. ≥ 3 complications, *n* (%)	0 (0)	6 (16.7)	< 0.01^a^
Postoperative hospital stay, days	14.3 ± 1.2	26.6 ± 3.1	< 0.01^a^
Medical cost, ×10^3^ USD	15.01 ± 0.87	14.62 ± 0.80	0.67

*Note:* Data are given as mean ± standard deviation or *n* (%).

^a^ Significant difference.

### 3.2 Study 2

**FIGURE 3:**
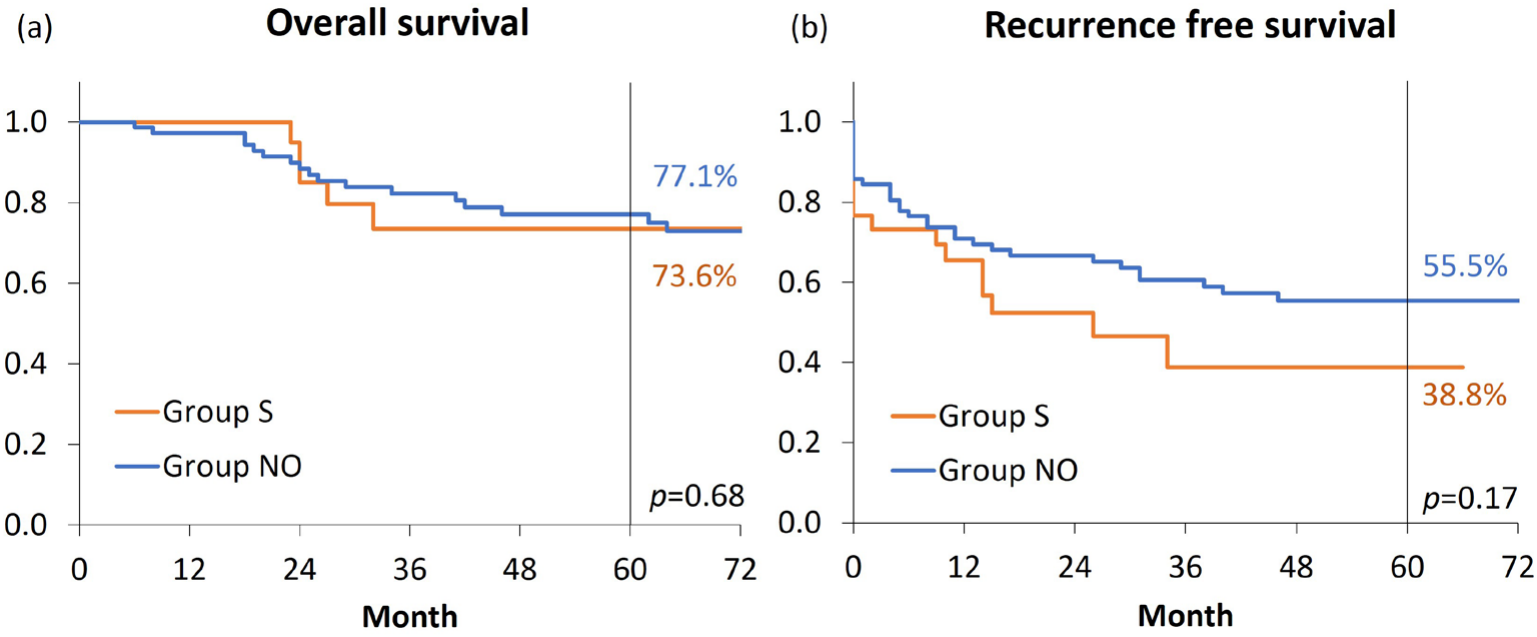
Kaplan–Meier analysis of Group S and Group NO. The 5‐year (a) overall survival rates (73.6% vs. 77.1%, *p* = 0.68; HR 1.40, 95% CI: 0.55–3.62) and (b) recurrence‐free survival rates (38.8% vs. 55.5%, *p* = 0.17; HR 1.51, 95% CI: 0.82–2.81) were not significantly different. Group S, patients who underwent self‐expandable metal stent placement; Group NO, patients selected using propensity score matching.

After matching, 77 patients were assigned to Group NO. The clinicopathological characteristics of both groups are summarized in Table 3. There were no significant differences in sex, age, PS, tumor location, or tumor stage between the two groups. Both groups included cases with R1/2 resection. Although there were no statistically significant differences in the rates of laparoscopic surgery or perioperative outcomes, the length of postoperative hospital stay was significantly shorter in Group S than in Group NO. The 5‐year postoperative overall survival (73.6% vs. 77.1%, *p* = 0.68) and 5‐year recurrence‐free survival rates (38.8% vs. 55.5%, *p* = 0.17) were not significantly different between Group S and Group NO (Figure 3). Recurrence occurred in 14 cases in Group S and in 33 cases in Group NO, and peritoneal dissemination recurrence occurred in five cases in both groups, with no significant difference (*p* = 0.12).

**TABLE 3:** Clinical characteristics and perioperative outcomes of patients in Group S and Group NO after propensity‐score matching.

	Group S, *n* = 30	Group NO, *n* = 77	*p*
Age, mean	72.2 ± 2.7	73 ± 0.9	0.68
Male, *n* (%)	20 (66.7)	49 (63.6)	0.83
PS, 0/1/2, *n*	25/5/0	64/13/0	1.00
Site, A/T/D/S/R, *n*	1/5/2/14/8	1/17/0/35/24	0.22
pT, 3/4, *n*	21/9	58/19	0.63
pN, 0/1/2/3, *n*	12/9/8/1	35/26/16/0	0.42
pM, 0/1, *n*	17/13	50/27	0.51
pStage, II/III/IV, *n*	10/7/13	26/24/27	0.69
R0/1/2, *n*	23/0/7	63/4/10	0.25
Laparoscopic surgery, *n* (%)	12 (40)	18 (23.4)	0.10
Operation time, min	326.6 ± 18.2	321.4 ± 17.2	0.24
Blood loss, mL	286.6 ± 59.8	249.8 ± 47.8	0.56
All grade complications, *n* (%)	17 (56.7)	31 (40.3)	0.14
Gr. ≥ 3 complications, *n* (%)	0 (0)	4 (5.2)	0.35
Postoperative hospital stay, days	14.3 ± 1.2	21.5 ± 1.3	< 0.01^a^

*Note:* Data are given as mean ± standard deviation or *n* (%).

Abbreviations: A, ascending colon; D, descending colon; PS, performance status; R, rectum; S, sigmoid colon; T, transverse colon.

^a^ Significant difference.

## 4 Discussion

The placement of colonic stents for OCRC was first reported in 1991, when a metal stent was placed for obstructive rectal cancer [8]. Since then, BTS has become prevalent worldwide, transitioning from its initial use in palliative care to being a method of preoperative BTS decompression for OCRC. SEMS placement has been reported to result in lower postoperative complication and mortality rates than emergency surgery, demonstrating favorable short‐term outcomes [9]. Recently, SEMS placement has gained attention as a BTS method to avoid emergency surgeries. In Japan, it has been covered by insurance since 2012 and has become routine practice in daily clinical care. However, the safety of the SEMS placement remains controversial. Although numerous studies have reported the superiority of SEMS over emergency surgery [6, 9, 10], comparative research on transanal decompression tubes remains limited.

A previous report from Japan indicated a perforation rate of 2% during SEMS placement [11]. In our study, two cases (6.7%) of perforation occurred in Group S. Although the perforation rate was slightly higher than that of the previous report, this was attributed to the limited number of cases, and the incidence was equivalent in Group T. This indicates that SEMS placement does not lead to a higher incidence of perforation. In terms of short‐term perioperative outcomes, the rate of laparoscopic surgery was significantly higher in Group S than in Group T (40% vs. 2.7%). This result is comparable with that of a previous report by Rodrigues‐Pinto et al., which compared SEMS placement with emergency surgery (43.8% vs. 2.2%) [12]. The higher proportion of minimally invasive laparoscopic surgeries in Group S may have contributed to the significantly lower intraoperative blood loss in that group. Regarding the degree of decompression, SEMS may have superior decompression efficiency because significantly more cases in Group S completed laparoscopic surgery and the time between decompression procedure and surgery was longer. According to a review by Matsuda et al., which compared SEMS and transanal decompression tubes, the postoperative complication rate for elective surgery after SEMS placement was 21.1% [13]. In the present study, the all‐grade complication rate of 60% according to the Clavien–Dindo classification might be considered high, but no grade ≥ 3 severe complications were observed in Group S. Comparatively, the incidence of severe complications in Group S was significantly lower than that in Group T (16.7%). This finding suggests a favorable short‐term outcome for SEMS, making it a valuable BTS strategy. Additionally, the lower occurrence of severe complications requiring extensive treatment contributed to shorter postoperative hospital stay. Despite the high costs associated with SEMS and laparoscopic surgery, the overall healthcare costs are believed to be controlled. Some reports have indicated that SEMS placement has been reported to result in greater improvement of pathological edema than transanal decompression tubes and thus may reduce colostomy placement [13]. In our study, the rate of stoma creation between groups S and T did not significantly differ, and we could not determine if the use of SEMS could avoid ostomies. The stoma formation rate was nearly equivalent to that reported by Kagami et al. (30.8%) [14]. In this study, CT to confirm the condition of the colon proximal to the obstruction site was not performed in all cases immediately before surgery; therefore, the degree of edema improvement was not examined. Previously, the decision to create a stoma was made at the discretion of the surgeon at our institution. However, SEMS placement followed by preoperative CT imaging, to assess the extent of improvement in colonic edema, in all cases may contribute to the evaluation of procedures aimed at avoiding stoma creation. Moreover, some reports suggest that immunological and nutritional markers in colorectal cancer can predict complications and prognosis [15, 16]. SEMS placement for OCRC allows oral intake and reduces the decrease in serum albumin levels compared to transanal tubes [17]. Another report indicated that preoperative nutritional status is associated with shorter postoperative hospital stays [18]. Although nutritional status was not assessed in this study, the lower incidence of complications and shorter postoperative hospital stay in Group S suggested a potential influence of nutritional status. Therefore, SEMS may be preferable to improve nutrition status.

Thus far, SEMS placement as a BTS has been recognized to improve short‐term perioperative outcomes. However, considering the oncological aspects, the impact on long‐term prognosis must be evaluated. Some reports have suggested that perforation during SEMS placement may increase recurrence rates [4]. The SEMS placement was designed to compress the tumor and secure the lumen. From our surgical perspective, this approach contradicts the idea of minimizing direct contact with the tumor during resection, whenever possible. Previous studies have reported an increase in perineural invasion in the group that underwent deferred surgery after stent placement as a BTS for colorectal cancer compared with the deferred surgery group for non‐OCRC [19]. Additionally, Kim et al. reported that the group undergoing deferred surgery after stent placement as a BTS had poorer overall and recurrence‐free survival rates than the non‐OCRC group [20]. The report suggested that the tumor's oncological impact was more substantial, considering the nature of the tumors leading to obstruction, rather than attributing it solely to the effect of stent placement. On the other hand, SEMS and transanal decompression tube have been reported to have no effect on long‐term prognosis [5, 6]. Therefore, in the present study, we compared the long‐term outcomes of SEMS with those of non‐OCRC using PSM to minimize bias. There were no significant differences in overall survival and recurrence‐free survival rates between Group S and Group NO.

The current study has several limitations. First, it was a retrospective observational study, and the study was conducted at a single institution, which led to a limited number of cases. Furthermore, the lack of a standardized strategy for SEMS or transanal tube placement and the subjective judgment of internists and surgeons at our hospital may have introduced bias in the case selection. In this study, the tumor invasion depth in Group S was milder than that in Group T, which may be an effect of bias. Additionally, the cost of SEMS placement was not reflected when the patients received interventions at another facility before starting treatment at our institution. Second, the observation period of this study was from 2004; however, insurance coverage for SEMS placement in Japan began in 2012. During this period, the treatment strategies for colorectal cancer have evolved dramatically. Treatment strategies may have differed between Group S and the other groups, and the observation period also differed, which may have affected the outcomes. Finally, in recent years, chemoradiotherapy and total neoadjuvant therapy have become increasingly common for advanced rectal cancer [21]. Facilities performing preoperative treatment after stoma creation for obstructive rectal cancer are expected to increase in the future. Therefore, a comparison between SEMS placement and stoma creation should be considered. Large‐scale prospective studies are necessary to further investigate these aspects.

## 5 Conclusions

The placement of SEMS as a BTS for OCRC, compared with ileus tubes, reduces postoperative complications and the length of hospital stay while maintaining comparable medical costs. Moreover, its impact on the long‐term prognosis was minimal. SEMS placement as a BTS is a valuable approach for the treatment of OCRC.

## Author Contributions

**Ryosuke Aoki:** conceptualization (lead), data curation (lead), investigation (equal), writing – original draft (lead). **Nao Hondo:** conceptualization (equal), investigation (lead), methodology (lead), writing – review and editing (lead). **Masato Kitazawa:** conceptualization (equal), data curation (equal), methodology (equal), supervision (lead), writing – review and editing (equal). **Satoshi Nakamura:** methodology (equal), supervision (equal), writing – review and editing (equal). **Makoto Koyama:** methodology (equal), supervision (equal), writing – review and editing (equal). **Masahiro Kataoka:** data curation (supporting). **Hirokazu Tanaka:** data curation (supporting). **Takuya Iguchi:** data curation (equal). **Yonghan Park:** data curation (equal). **Yuji Soejima:** conceptualization (equal), methodology (equal), supervision (equal), writing – review and editing (supporting).

## Disclosure

The authors have nothing to report.

## Ethics Statement

The study protocol for this research project was approved by the Institutional Ethics Committee of Shinshu University and conformed to the provisions of the Declaration of Helsinki (approval number: 5691). This study was conducted using an opt‐out approach, with the approval of the Ethics Committee, in accordance with the *Ethical Guidelines for Medical and Biological Research Involving Human Subjects* (2021, issued by the Ministry of Education, Culture, Sports, Science and Technology and the Ministry of Health, Labour and Welfare and the Ministry of Economy, Trade and Industry of Japan). As the study used only existing data and did not involve any invasive procedures or collection of new biological specimens, the requirement for obtaining written informed consent was waived in accordance with Chapter 4, Section 8‐1 of the guidelines.

## Conflicts of Interest

The authors declare no conflicts of interest.

## Data Availability

The data that support the findings of this study are not publicly available due to patient privacy concerns but are available from the corresponding author on reasonable request and with approval of the institutional review board.
